# Response to pegylated interferon in a COVID‐19–positive elderly woman with primary myelofibrosis treated with ruxolitinib

**DOI:** 10.1002/ccr3.3997

**Published:** 2021-03-07

**Authors:** Arthur E. Frankel, Renuka Reddy, Kayla R. DeSuza, Khaled Deeb, Aaron F. Carlin, Davey Smith, Yushuang Xie, Eknath Naik, Richard T. Silver, Hans C. Hasselbalch

**Affiliations:** ^1^ Department of Medicine West Palm Beach VA Medical Center West Palm Beach FL USA; ^2^ Division of Infectious Disease UCSD Medical School San Diego CA USA; ^3^ Division of Hematology and Medical Oncology Weill Cornell Medicine New York NY USA; ^4^ Department of Hematology Zealand University Hospital Roskilde Denmark

**Keywords:** COVID‐19, interferon, ruxolitinib

## Abstract

An 83‐year‐old female had asymptomatic SARS‐CoV‐2 infection while taking ruxolitinib. She remained RT‐PCR positive for viral RNA for >120 days, and Pegylated interferon for 4 weeks led to viral RNA clearance. The observations support combination therapy of ruxolitinib + interferon for COVID‐19.

## Graphical abstract

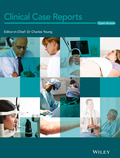

## 1 INTRODUCTION

SARS‐CoV‐2 is a positive‐sense, single‐stranded RNA in the *Coronaviridae* family of viruses.^1^, ^2^ Most cases of infection present with mild disease phenotype with self‐limiting symptoms largely consist of fever, fatigue, dry cough, headache, and diarrhea.^1^, ^2^ However, roughly 14% of patients develop a severe disease phenotype requiring hospitalization, most commonly due to dyspnea and hypoxia.^3^, ^4^ Characteristic laboratory features of this virus are leukopenia, prolonged prothrombin time, and elevated serum concentrations of D‐dimer, lactate dehydrogenase (LDH), ferritin, and C‐reactive protein (CRP).^5^ Chest‐computed tomography classically demonstrates bilateral ground‐glass opacities.^1^ A critical component of the pathogenesis of SARS‐CoV‐2 consists of a hyperactive immune response to the virus resulting in a sudden, acute increase in pro‐inflammatory cytokines, termed “the cytokine storm”.^6^ Key pro‐inflammatory cytokines upregulated in this process include interleukin 6 (IL‐6) and tumor necrosis factor‐alpha (TNF‐α).^6^ IL‐6 is one of the most highly expressed cytokines in SARS‐CoV‐2 infection, and elevated serum levels of IL‐6 are associated with a poor prognosis.^7^, ^8^, ^9^ Elevated cytokine levels prompt an influx of various immune cells into the site of infection, leading to tissue destruction and multiorgan damage.^10^ Immune‐mediated tissue destruction is thought to be a contributing factor to the development of several life‐threatening complications, such as acute respiratory distress syndrome, septic shock, and multiorgan failure.^11^

Primary myelofibrosis (PMF) is an acquired stem cell neoplasm with ineffective hematopoiesis, bone marrow fibrosis, and splenomegaly. Clonal populations of dysplastic megakaryocytes and myeloid cells release inflammatory cytokines that are responsible for clonal evolution, symptom burden, progressive myelofibrosis, and extramedullary splenic hematopoiesis.^12^ Common gene mutations found in patients with PMF include Janus kinase 2 (JAK2V617F), calreticulin (CALR), thrombopoietin receptor (MPL515L/K), and ten‐eleven translocation 2 (TET2). The early phase of PMF is termed the prefibrotic phase when patients have traditionally been thought to be asymptomatic. This phase is characterized by a hypercellular marrow with megakaryocytic hyperplasia and minimal fibrosis.^12^ At later stages of disease progression, myelofibrosis develops due to the release of excessive amounts of growth factors from megakaryocytes and monocytes resulting in fibroblast proliferation, collagen synthesis, and an increasing degree of fibrosis.^12^ Patients may express cytokine‐related hypercatabolic symptoms such as fatigue, weight loss, fever, and chills, together with abdominal discomfort from splenomegaly. Splenomegaly is primarily due to extramedullary hematopoiesis but can also be due to splanchnic vein thrombosis.^12^ Treatment largely depends on disease burden; while many patients are observed without treatment, young, high‐risk patients may receive allogenic stem cell transplant.^13^ For other patients, therapies are designed to dampen the excessive inflammatory marrow milieu.^14^ On the molecular level, Janus kinases (JAKs) mediate cytokine production through various downstream signaling pathways, such as the signal transducer and activator of transcription (STAT) pathway.^15^ The JAKs consist of four tyrosine kinases that consist of JAK1, JAK2, JAK3, and tyrosine kinase 2 (TYK2). They transmit extracellular signals, such as pro‐inflammatory cytokines, to the nucleus by activating STAT. Ultimately, these extracellular signals result in a transcriptional response of target genes from cellular DNA.^16^ In this pathway, receptors bind to various cytokines that, in turn, trigger and orchestrate innate immune responses.^17^ Additionally, interferon acts through the JAK‐STAT pathway to target genes responsible for antiviral and adaptive immune responses.^18^ The JAK2V617F variant, a gain‐of‐function mutation, causes constitutive activation of tyrosine kinase domain of JAK2 leading to dysregulated immune response.^19^ This mutation is present in majority of patients with myeloproliferative neoplasms. JAK inhibitors ruxolitinib and fedratinib are FDA approved for the treatment of PMF.^20^ The immunosuppressive effects of JAK inhibition vary based on the specificity and dosage of each drug, which also accounts for the range in toxicology profiles. Ruxolitinib is a JAK 1‐2 inhibitor that causes a reduction in cytokine production. This drug was shown to decrease spleen size and disease‐related symptoms compared with placebo in the double‐blind COMFORT‐I trial consisting of 309 patients with intermediate‐2 or high‐risk myelofibrosis. Ruxolitinib is primarily utilized in the treatment of myelofibrosis, but it is also licensed for patients with polycythemia vera intolerant or refractory to hydroxyurea. However, ruxolitinib is also utilized off‐label for diseases involving cytokine release as the primary pathogenesis, including graft‐vs‐host disease and hemophagocytic lymphohistiocytosis.^21^, ^22^ Multiple small‐molecule JAK inhibitors are also utilized in the treatment of many inflammation‐driven pathologies, such as inflammatory bowel disease, rheumatoid arthritis, and psoriasis.^23^ Another molecular component in inflammation regulation is interferon which for decades has been used successfully in the treatment of patients with myeloproliferative neoplasms. Interferon normalizes cell counts in the majority of patients within a few months. Interferon also improves megakaryocytic dysfunction in part through induction of IFITM3.^24^ This led to the treatment of early phase PMF with interferon.^25^, ^26^, ^27^ Eighty percent stabilization, partial response, or remission was observed in phase 2 studies. Architectural reversion of the marrow fibrosis after treatment was noted. Recent studies have demonstrated efficacy of ruxolitinib and interferon α2 combination PMF treatment with an acceptable toxicity profile.^28^ Combination therapy was shown to elicit complete remissions in 3 out of 18 patients and complete hematologic response in 11 out of 12 patients.^29^

Janus kinase/STAT pathway inhibitors have been proposed as a therapy to target the hyperinflammation associated with SARS‐CoV‐2.^15^, ^30^ This hyperinflammation seen in SARS‐CoV‐2 is similarly observed in cytokine release syndrome (CRS), characterized by elevated IL‐6, IL‐2, IL‐7, IL‐10, and more.^1^, ^31^ Elevations in serum cytokine and chemokine levels correlate with disease severity and adverse clinical outcome.^1^ Specifically, increased levels of IL‐6 have been reported in patients with severe SARS‐CoV‐2 and have been associated with increased mortality.^7^, ^8^, ^9^ IL‐6 plays pivotal role in CRS through JAK‐STAT signaling that results in altered immune regulation and oxidative stress.^16^ Therefore, many treatments are aimed at ameliorating the cytokine storm by inhibiting the JAK‐STAT pathway. Ruxolitinib has been shown to significantly reduce IL‐6 and CRP levels in patients with myelofibrosis, with a relatively mild side effect profile and is therefore being considered as a treatment option for SARS‐CoV‐2.^32^ Of note, there is concern for increased risk of infection in patients treated with JAK inhibitors, as JAK‐STAT signaling is responsible for the signal transduction of type I interferon.^16^ Interferons are crucial for preventing viral replication in the early stage of infection in addition to enhancing antibacterial immunity.^33^ This was evidenced by previous studies reporting increased incidence of bacterial infections, particularly urinary tract infections in patients treated with JAK inhibitors.^34^ Interferons may be protective early in SARS‐CoV‐2 infection and damaging later in the infection. Thus, the effects of interferon in COVID‐19 patients are likely complex and time dependent.

We present the case of an 83‐year‐old woman found to be SARS‐CoV‐2 positive who was asymptomatic while taking ruxolitinib for co‐existing PMF but displayed a prolonged period of nasal swab PCR positivity. Culture failed to reveal infectious virions. Administration of pegylated interferon was followed by rapid clearance of viral RNA by PCR. We hypothesize that the combination of ruxolitinib with interferon may be useful in the acute COVID‐19 setting to induce viral clearance with reduced risk of cytokine storm.

## 2 METHODS

Informed consent to monitor, treat, and report the subject was obtained from the family and approved by the West Palm Beach VA Research & Education Committee. Nasopharyngeal swab specimens were obtained from the patient. Samples were shipped to Orlando for performance of the Gene Xpert Cepheid Innovation XPRSARS‐COv2‐10 RT‐PCR assay measuring viral N2 sequence abundance. The limit of detection was 0.01 plaque‐forming units/mL (CFU/mL) or 250 viral RNA copies/mL with first cycle number above background (Cq) of 39. Blood samples were collected using a serum separator tube and serum also shipped to Orlando for performance of the Abbott Architect anti‐N SARS‐CoV‐2 IgG assay with results calculated as chemiluminescence ratios of sample to control with negative ratios being <1.4. Additional nasopharyngeal swab sample was shipped on dry ice to University of California San Diego for viral culture in a Biosafety Level‐3 facility. For measurement of infectious virus, Vero E6 was obtained from American Type Culture Collection, Rockville, MD and grown in Dulbecco's minimal essential medium (DMEM, Corning) with 10% fetal calf serum and penicillin‐streptomycin (Gibco). The clinical sample was thawed, and 200uL per well was added to row A (column 2‐12) of a 96‐well plate. Then, 100ul of serum‐free DMEM was added to rows B‐H and clinical samples were twofold serially diluted by transferring 100ul down the rows of the plate (B‐H). Then, the entire volume of each well was transferred to a 96‐well plate containing 20,000 Vero E6 cells per well in 100ul of DMEM, 10% fetal bovine serum, 2 × penicillin‐streptomycin, 2 × antibiotic‐antimycotic, and 2 × amphotericin B (Gibco) and gently mixed. Inoculated cultures were grown in a humidified 37°C incubator with 5% CO_2_ and observed for cytopathic effects (CPEs) for a total of 6 days. No CPE was observed after 6 days, and thus, the samples were blind passaged onto a fresh 96‐well plate containing 20 000 Vero E6 cells per well and cultured at 37°C with 5% CO_2_ and observed for CPE for an additional 6 days. Infectious SARS‐CoV‐2 produces CPE on Vero E6 cells. All work with potentially infectious SARS‐CoV‐2 clinical material was conducted in Biosafety Level‐3 conditions at the University of California San Diego following guidelines approved by the Institutional Biosafety Committee.

## 3 RESULTS/CASE PRESENTATION

An 83‐year‐old Puerto Rican woman presented to the emergency department from a skilled nursing facility owing to generalized fatigue, weakness, and mechanical falls. On admission, the patient denied fever, chills, shortness of breath, chest pain, cough, nor sputum production. She was afebrile and hypotensive with a blood pressure of 90/50 mm Hg. On physical examination, she appeared thin and chronically ill. She was alert and oriented only to self, which was reportedly her baseline state. Her initial laboratory work was remarkable for a hemoglobin 6.0 g/dL, mean corpuscular volume of 95.3 fL, white blood cell (WBC) count 5.8K/µL, and platelet count 351 K/µL. Her creatinine level of 2.50 mg/dL was elevated from her baseline of 1.30 mg/dL. Her past medical history was remarkable for dementia, primary myelofibrosis with macrocytic anemia and thrombocytosis, hypertension, chronic kidney disease stage III, and osteoporosis. She had a remote history of 40 pack‐years cigarettes. She had no history of chemotherapy, radiation, or chemical exposures. In October 2015, the patient was diagnosed with JAK2V617F‐positive PMF. Cytogenetic analysis revealed a normal karyotype. From diagnosis, the patient was treated with hydroxyurea 500 mg daily and aspirin 81 mg daily. She was dependent upon monthly packed red blood cell transfusions. Four months prior to hospitalization, treatment with ruxolitinib 20 mg daily was started.

Her admitting diagnosis was anemia secondary to primary myelofibrosis and acute kidney injury. She was transfused with two units of packed red blood cells. The following morning, she developed a mild nonproductive cough and a fever of 101.2°F. Her oxygen saturation ranged between 90% and 92% on 2 L of nasal cannula. Using a nasopharyngeal swab, the patient tested positive for SARS‐CoV‐2 by RT‐PCR. RT‐PCR results during her hospitalization are displayed in Table 1. Chest radiograph was unremarkable; procalcitonin was normal at 0.09 ng/mL. The following inflammatory markers were elevated: CRP 3.41 mg/dL, D‐dimer 258 ng/mL, ferritin 1329 ng/mL, LDH 739 U/L, and fibrinogen 769 mg/dL (see Table 2). The patient was subsequently treated with hydroxychloroquine and azithromycin. Although the patient was largely asymptomatic, she remained an inpatient for the next 4 weeks as she was unable to be discharged back to her nursing home owing to persistently positive SARS‐CoV‐2 tests. Her persistently positive tests were attributed to immunosuppression from ruxolitinib causing the patient to have impaired viral RNA clearance. Nasal swab was tested for infectious virus by cytopathic effect in mammalian cell tissue culture (see Methods). No infectious virions were found. After discussing the risks and benefits with her family, ruxolitinib was discontinued. One day after stopping ruxolitinib, the patient developed a low‐grade fever of 101.1°F and became hypoxic. Oxygen saturation ranged between 90% and 92% on room air. Her creatinine increased from 1.30 mg/d to 1.50 mg/d with leukocytosis ranging from 11 K/uL to 17 K/uL and thrombocytosis ranging from 450 K/uL to 550 K/uL. During this time, d‐dimer, the only inflammatory marker measured, increased from 258 ng/mL on admission to 329 ng/mL, as depicted in Table 2. Chest radiograph was still unrevealing. Four days after stopping ruxolitinib, the patient was lethargic with increased urinary frequency. Subsequently, she was found to have a urine culture growing *Enterococcus faecalis* and she was treated with intravenous ampicillin and transitioned to oral amoxicillin for a total of 7 days of treatment. The patient did not receive ruxolitinib for 5 days total. We were concerned about JAK withdrawal syndrome secondary to JAK2 activation loop phosphorylation^35^ vs worsening SARS‐CoV‐2 and committed to read ruxolitinib. After restarting ruxolitinib, the patient rapidly improved and was no longer febrile or hypoxic. Her WBC count decreased to 7.8‐9.0 K/uL and platelet counts decreased to 424‐540 K/uL. D‐dimer was not re‐measured. Following this brief drug holiday, the patient remained positive for SARS‐CoV‐2 by RT‐PCR testing. She remained in isolation. She required two blood transfusions over the following 2 months. The decision to stop ruxolitinib for a second time was made in an attempt to give her immune system a second chance of eliminating the viral RNA. This time, the patient developed a mild cough with a leukocytosis ranging from 12.0 to 17.4 K/uL. Her platelet counts remained within normal limits. Ruxolitinib was discontinued for 6 days total and re‐initiated due to the patient's worsening clinical status. Despite this second trial off ruxolitinib, she was unable to achieve a negative PCR. Due to her persistently positive SARS‐CoV‐2 testing, the patient was given a 45‐mcg subcutaneous injection of pegylated interferon α2a approved by the Veterans Affairs Hospital in hopes of facilitating viral RNA clearance. Three days following pegylated interferon administration, her RT‐PCR test was still positive for SARS‐CoV‐2, and the patient was given weekly doses for a total of four doses. Ten days after receiving the second dose of pegylated interferon, the patient had a negative RT‐PCR test. The patient did not experience any side effects from the pegylated interferon treatment. The patient ultimately cleared the viral RNA from nasal swabs on treatment with ruxolitinib with subcutaneous pegylated interferon (Table 1). Anti‐N SARS‐CoV‐2 IgG antibodies were maximal early at Day 50 of hospitalization and fell progressively after 2 months similar to the reported anti‐N IgG half‐life of 35 days^36^ (Table 3).

**TABLE 1:** RT‐PCR Cq values during hospitalization^a^

Day	Quantification cycle (Cq)	Test result
2	27.9	Positive
55	40.6	Positive
68	36.1	Positive
78	41.0	Positive
88	40.1	Positive
93	42.5	Positive
98	35.7	Positive
99	42.0	Positive
110	41.7	Positive
117	N/A	Negative
124	40.1	Positive
131	N/A	Negative
134	N/A	Negative
139	39.0	Positive
146	N/A	Negative
154	N/A	Negative
181	N/A	Negative

^a^ RT‐PCR Cq, reverse transcriptase‐polymerase chain reaction cycle at which fluorescence detected above baseline. The value is inversely related to quantity of viral RNA.

**TABLE 2:** Inflammatory markers during hospitalization^a^

Day	Markers	Value	Normal range
98	Interleukin 2	<38	100‐500 u/mL
110	Interleukin 6	10.51 pg/mL	<5.00 pg/mL
110	Ferritin	>1675.56 ng/mL	10‐380 ng/mL
110	LDH	523 U/L	100‐230 U/L
110	C‐reactive protein	0.47 mg/dL	0.00‐1.00 mg/dL
110	D‐Dimer	<150 ng/mL	≤229 ng/mL
111	Interleukin‐2Rα/CD25	1886 pg/mL	532‐1891 pg/mL
119	Interleukin 10	0.6 pg/mL	4.8‐9.8 pg/mL
119	Interleukin‐2Rα/CD25	2038 pg/mL	532‐1891 pg/mL
119	Interleukin 6	15.03 pg/mL	<5.00 pg/mL
141	SARS‐CoV‐2 IgG Anti‐N	Negative	
141	SARS‐CoV‐2 IgG Anti‐N	1.18 S/Co	0.00‐1.39 S/Co

^a^ LDH, lactate dehydrogenase; SARS‐CoV‐2, severe acute respiratory syndrome coronavirus‐2; IgG, immunoglobulin G.

**TABLE 3:** Anti‐N SARS‐CoV‐2 IgG antibody titers during hospitalization^a^

Day	Titer	Test result
50	5.76	Positive
88	3.79	Positive
179	1.18	Negative
229	0.58	Negative
255	0.52	Negative

^a^ Negative range is 0‐1.39; N is nucleocapsid protein; Abbott Architect anti‐N SARS‐CoV‐2 IgG assay.

## 4 DISCUSSION

SARS‐CoV‐2 is associated with a wide range of symptoms ranging from a mild clinical phenotype with fever and cough to severe respiratory and/or multiorgan failure. SARS‐CoV‐2 has considerable morbidity and mortality, particularly among people with advanced age and comorbities.^37^ A significant factor contributing to the morbidity and mortality of this infection is the pulmonary and systemic inflammatory response.^38^ Multiple SARS‐CoV‐2 proteins and viral RNAs trigger inflammation. Endosomal and cytoplasmic viral RNA binds TLR and NOD pathway receptors^38^; ORF3a, ORF3b, ORF7a, ORF8a, ORF9b, and E envelope proteins are pro‐apoptotic, release NF‐κB or activate the NLRP3 inflammasome.^39^, ^40^ Subsequently, inflammasome caspases cleave interferon signal pathway components cGAS, MAVS, and IRF3 blocking antiviral interferon responses at the same time as the marked inflammatory reaction.^41^

We were struck by the minimal clinical findings in this high‐risk, elderly woman with a co‐existing hematopoietic malignancy. Our patient was on chronic ruxolitinib therapy for myelofibrosis. Ruxolitinib inhibits JAKs and TYK2 and thus downstream STATs and cytokine expression in T lymphocytes, neutrophils, and dendritic cells.^42^ We speculate whether our patient's minimal clinical symptoms throughout her infection could be linked to the immunosuppressive effect of the drug. Ruxolitinib may reduce the SARS‐CoV‐2 inflammatory state, improve the quality of life, and perhaps prolong survival from this devastating disease. This speculation is supported by several pilot studies. A trial by Giudice et al demonstrated a significant improvement in respiratory symptoms and radiographic pulmonary lesions in seven SARS‐CoV‐2 patients with acute respiratory distress syndrome treated with a combination of ruxolitinib and eculizumab, an anti‐C5a complement monoclonal antibody.^43^ A retrospective study by La Rosee et al showed ≥25% reduction in COVID‐19 Inflammation Scores (CIS) after 7 days of treatment with ruxolitinib in a subset of 14 patients with CIS ≥10.^44^ The CIS score measured chest X‐ray abnormalities, levels of CRP, ferritin, triglycerides, IL6, fibrinogen, blood white cell count, blood lymphocyte count, d‐dimer, PTT, and presence or absence of fever. Moreover, Cao et al conducted a multicenter, randomized control trial evaluating the efficacy of ruxolitinib in 43 patients with severe SARS‐CoV‐2 infection. Ruxolitinib recipients showed a significant improvement in chest‐computed tomography and faster recovery from lymphopenia compared with the control group.^45^ This trial also revealed that ruxolitinib was well tolerated with infrequent toxicities.^30^ Theoretically, higher rates of aberrant JAK 2 activating mutations in older myeloproliferative neoplasm patients could enhance the hyperinflammatory state induced by SARS‐CoV‐2.^33^ Nevertheless, treatment with ruxolitinib should proceed cautiously as ruxolitinib and SARS‐CoV‐2 have both been associated with coagulopathy and increased frequency of thromboembolic events.^46^

An interesting facet of this case is the sustained positivity of the patient's SARS‐CoV‐2 test. She was repeatedly tested for viral RNA clearance by nasal swab RT‐PCR secondary to her immunocompromised state and because she required a negative test prior to discharge to her nursing facility. Many SARS‐CoV‐2–infected individuals have persistently positive RT‐PCR tests for weeks to months after clinical recovery.^47^ Based on viral culture, the percent of these individuals who remain infectious approaches zero by 10‐15 days after the onset of symptoms.^47^, ^48^, ^49^ However, shedding of infectious SARS‐CoV‐2 has been demonstrated by viral culture or inferred by the presence of subgenomic RNA in a subset of individuals, including immunosuppressed hosts, for months following infection.^50^, ^51^ Higher Cq values of SARS‐CoV‐2 RT‐PCR reflect lower viral loads, and multiple studies have demonstrated inability to culture infectious virus above certain Cq thresholds.^48^ As demonstrated in Table 1, the Cq of the ten subsequent RT‐PCR samples by nasopharyngeal swab ranged from 35.7 to 42.5 with a mean of 38.2. Based on the referenced literature, these values likely represent the presence of low quantities of viral RNA (vRNA) or vRNA fragments that are noninfectious, although the Cq thresholds are not directly comparable across assays. We were unable to culture infectious virus from our patient at day 98. However, it should be noted that respiratory viral culture is insensitive, and lack of viral growth in vitro does not ensure lack of infectiousness.

The persistent positivity of her SARS‐CoV‐2 testing may be potentially secondary to the immunosuppressive effective of the ruxolitinib.^34^ Ruxolitinib targets components of both the innate and adaptive immune systems. JAK/TYR2 proteins are downstream for both innate immune cytokines and adaptive immune interferon receptors.^52^ Therefore, suppression of the pathway places a person susceptible to various infections.^22^ With these defense mechanisms impaired, the drug contributes to increased risk of reactivation of silent viral, bacterial, and fungal infections.^53^, ^54^ This viral susceptibility is due to JAK/TYR2 inhibitors suppressing cytokines, such as interferon, and NK cells.^22^ This case addresses the issue of hampered antiviral defense caused by ruxolitinib through the supplementation of interferon with subsequent T‐cell activation to fight SARS‐CoV‐2 infection. Our patient was able to clear the vRNA approximately 30 days after the administration of a total of four treatments of pegylated interferon‐α2a while continuing treatment with ruxolitinib.

Viruses such as SARS‐CoV‐2 have evolved to facilitate their own infectivity and to evade host detection and immune response. SARS‐CoV‐2 activation of pro‐inflammatory pathways described above^39^, ^55^, ^56^ generates intracellular caspases that degrade interferon and interferon signaling polypeptides.^41^ Previous data on SARS‐CoV and MERS‐CoV outbreaks have revealed additional mechanisms of coronavirus type I interferon suppression.^46^, ^56^, ^57^, ^58^ To date, data exist showing that 12 of the 29 SARS‐CoV‐2 proteins block IFN production early: nsp1 inhibits 40S ribosome participation in IFN translation; nsp3 blocks RIG‐1 PAMP signaling; nsp10 performs 2‐O‐methyltransferase cap on vRNA to hide the vRNA; nsp13 binds and inhibits TBK1 PAMP signaling; nsp14 performs N7‐methyltransferase caps on vRNA again to disguise the virus; nsp15 removes 5'pU tracts from vRNA to avoid vRNA detection; nsp16 assists in 2‐O‐methyltransferase cap formation on vRNA; ORF3b binds and blocks IRF3 signaling; ORF6 inhibits karyopherin so cytoplasmic to nuclear PAMP signaling is blocked; M protein binds and blocks TRAF/TBK1 signaling; orf9b binds and blocks MAVS PAMP signaling; and N protein binds and blocks RIGI PAMP signaling. SARS‐CoV‐2 produces a delayed first‐line antiviral defense followed by excessive inflammatory cytokinemia and dysfunctional T‐ and NK cell responses.^33^, ^46^

Interferons have been successfully used in the treatment of viral infections, such as hepatitis C, autoimmune diseases such as multiple sclerosis, and hematologic malignancies such as essential thrombocythemia, polycythemia vera, and myelofibrosis.^59^, ^60^, ^61^ In SARS‐CoV‐2, interferon therapy in phase 2 and phase 3 randomized clinical trials has shown reduced the duration of virus infection, reduced inflammatory markers including IL6 and CRP, and reduced mortality when administered early.^56^, ^62^, ^63^, ^64^, ^65^, ^66^, ^67^, ^68^ As a note of caution, type I interferons administered in later stages may cause progressive tissue damage leading to a deleterious hyperinflammation characterized by the excessive macrophage activation and hypercoagulation seen in patients with acute disease.^38^ Interestingly, pharmacologic interferon treatment inhibits inflammation early by repressing the NLRP3 inflammasome via STAT1 and STAT3.^69^ We hypothesized that administration of interferon in our patient who was minimally symptomatic would strengthen antiviral defense and potentially lead to viral RNA clearance. Our results support the hypothesis.

## 5 CONCLUSION

The availability of vaccines will reduce the number of acute cases of COVID‐19. However, acute cases will continue to exist, requiring therapeutic interventions to reduce toxicities and improve survival. Temporizing the cytokine storm appears to be crucial in preventing end‐organ damage which is associated with high mortality.^70^ Genetic and immunologic studies of hospitalized COVID‐19 subjects showed mutations yielding increased TYK2 or decreased IFNAR2 expression or inactivating mutations in interferon pathway genes—IRF3, IRF7, IFNAR1/2, TBK1, or TLR3 or autoantibodies to interferons had more severe disease.^71^, ^72^, ^73^ These subjects suffered increased inflammatory cytokines and absent antiviral interferons. Targeted immune regulation to reverse this state may provide substantial benefit in SARS‐CoV‐2 infection. Our case suggests that ruxolitinib plus pegylated interferon is a potential regimen for SARS‐CoV‐2 patients. This treatment combination may benefit select patients if used early in the disease. Future studies are needed to elucidate the potential therapeutic benefits and side effects of this regimen.

## CONFLICT OF INTEREST

None of the authors has a relevant conflict of interest.

## AUTHOR CONTRIBUTIONS

AF: hematologist of the patient, initiated the project, obtained institutional and subject approval for the treatments, obtained foundation support for interferon, and revised and oversaw the manuscript. RR: initiated the manuscript write up. KD: collaborated on the manuscript write up. AC: performed viral culture and reviewed manuscript. DS: oversaw culture work and reviewed manuscript. KD: was responsible for collecting RT‐PCR data. YX: was the hospitalist taking care of the patient while inpatient and was responsible for placing lab orders. EN: emergency room physician, assisted in data collection and review. HH: hematologist, proposed use of interferon in this subject and reviewed the work. RS: hematologist, also proposed interferon and participated in manuscript preparation.

## INFORMED CONSENT

The study was conducted in accordance with the Declaration of Helsinki. Informed consent was obtained for the interferon therapy and case report.
